# Effect of Mechanical Loads on Stability of Nanodomains in Ferroelectric Ultrathin Films: Towards Flexible Erasing of the Non-Volatile Memories

**DOI:** 10.1038/srep05339

**Published:** 2014-06-18

**Authors:** W. J. Chen, Yue Zheng, W. M. Xiong, Xue Feng, Biao Wang, Ying Wang

**Affiliations:** 1 State Key Laboratory of Optoelectronic Materials and Technologies, School of Physics and Engineering, Sun Yat-sen University, Guangzhou 510275, China; 2 Micro&Nano Physics and Mechanics Research Laboratory, School of Physics and Engineering, Sun Yat-sen University, Guangzhou 510275, China; 3 AML, Department of Engineering Mechanics, Tsinghua University, Beijing 100084, China

## Abstract

Intensive investigations have been drawn on nanoscale ferroelectrics for their prospective applications such as developing memory devices. In contrast with the commonly used electrical means to process (i.e., read, write or erase) the information carried by ferroelectric domains, at present, mechanisms of non-electrical processing ferroelectric domains are relatively lacking. Here we make a systematical investigation on the stability of 180° cylindrical domains in ferroelectric nanofilms subjected to macroscopic mechanical loads, and explore the possibility of mechanical erasing. Effects of domain size, film thickness, temperature and different mechanical loads, including uniform strain, cylindrical bending and wavy bending, have been revealed. It is found that the stability of a cylindrical domain depends on its radius, temperature and film thickness. More importantly, mechanical loads have great controllability on the stability of cylindrical domains, with the critical radius nonlinearly sensitive to both strain and strain gradient. This indicates that erasing cylindrical domain can be achieved by changing the strain state of nanofilm. Based on the calculated phase diagrams, we successfully simulate several mechanical erasing processes on 4 × 4 bits memory devices. Our study sheds light on prospective device applications of ferroelectrics involving mechanical loads, such as flexible memory devices and other micro-electromechanical systems.

Ferroelectrics are natural candidates for data storage, as they possess switchable spontaneous polarization to carry bit information “0” and “1”. Their usefulness also originates from a wide spectrum of other important properties, such as piezoelectricity, pyroelectricity, photovoltaic effect, and nonlinear optic behaviors^1^,^2^. Compared with semiconductors and magnetic materials that are predominately used in current storage technology, ferroelectrics are more attractive in developing memory devices, for their high-density storage capacity and the nonvolatility to retain information without power^3^. From an ideal point of view, based on scanning probe microscopy (SPM), Tb/inch^2^ ultrahigh-density storage can be achieved by forming ferroelectric nanodomains (~10 nm)^4^,^5^,^6^,^7^,^8^,^9^. By exploiting the influence of polarization on transport property, polarization-tunable effects, e.g., giant electroresistance (GER)^10^, giant piezoelectric resistance (GPR)^11^ and photovoltaic diode effects (PVD)^12^, are promising for non-destructive readout of domains. Recent progresses in experimental techniques bring the research of ferroelectrics to an entirely new level, with high-quality structures being realized, characterized and manipulated in atomic level^4^,^5^,^6^,^7^,^8^,^9^,^13^,^14^. Strategies for integrating ferroelectric structures on elastomeric supports also indicate the feasibility of flexible ferroelectric devices^15^,^16^. All these describe a tempting future of ferroelectric memory devices — high-density, nonvolatile, long standing and ultimately, flexible.

Nevertheless, at present there are still many challenges towards such a wonderful picture. The crucial issues include nanodomain stability and proper processing (i.e., reading, writing or erasing) mechanisms. In general, stability of a ferroelectric domain intrinsically depends on its size due to the collective nature. It is also sensitive to the environments surrounding it. Particularly for a ferroelectric nanofilm, its strain state and properties in the vicinity of surfaces/interfaces, e.g., defects, bondings and screening condition, can significantly affect the domain structure and thus the stability of specific domains^17^,^18^,^19^,^20^,^21^. Loading history, e.g., repetitive processing, also has great impact on the domain stability of the nanofilm. For example, the film gradually loses switchable domains after repetitive electrical switching, known as fatigue problem, due to the happening of electric-driven processes such as defect generation^22^. For the above complicated factors, although quite an amount of relevant works can be found in literature, stability of ferroelectric domains still needs further investigations to achieve an effective and quantitive control on it. For the simplest domain pattern for memory applications, i.e., 180° nanodomains, previous researches paid attention to dependence of their stability on SPM electric field (e.g., strength and period)^4^,^5^,^6^,^7^,^8^,^9^. The minimal domain size that can be achieved in SPM experiments so far is limited by the radius curvature of SPM tip (typically 20–50 nm)^9^. To reach a lower spatial limit of domain stability, exploiting the effects of other factors, such as mechanical loads would be beneficial.

Due to the natural coupling between polarization and electric field, almost all reported reading/writing/erasing mechanisms of ferroelectric domains are electrical. In literature, it has been understood that film-substrate mismatch strains can be utilized to obtain novel ferroelectric phases and domain structures in ferroelectric thin films^17^,^23^,^24^. However, few attentions have been paid to the evolution of an existed domain pattern in response to mechanical loads (i.e., treating mechanical loads in a similar role of electric field). Being a phenomenon originated from the collective distortions of lattice unitcells, ferroelectric polarization is intrinsically coupled with mechanical field. Consequently, external strain can change the polarization state, known as piezoelectricity. It has been also realized that strain gradient can affect polarization in a similar way of electric field, namely flexoelectricity (see reviewed paper Ref. 25). These electromechanical effects provide the possibility of processing ferroelectric domains mechanically. Indeed, by exploiting the flexoelectric field of local strain gradient, mechanical writing ferroelectric domains has been recently demonstrated^26^. Considering the possible problems of electrical means (e.g., leakage, heat, dielectric breakdown and fatigue), and potential applications of ferroelectrics in non-electrical environments, effective strategies for processing ferroelectric domains by mechanical means would be meaningful.

In this letter, based on systematical phase field simulations, we demonstrate that stability of polar domains in ferroelectric ultrathin films can be effectively controlled by macroscopic mechanical loads. As a consequence, ultrahigh density of stable polar domains can be obtained. Moreover, erasing of domains can be realized by adjusting the strain state of the nanofilm. With carefully taken into account the effects of inhomogeneous electromechanical fields, ambient temperature and surfaces, stability of 180° cylindrical domains has been comprehensively revealed as a function of domain size, temperature, film thickness and particularly various mechanical loads, such as uniform strain, cylindrical bending and wavy bending. The revealed mechanical controllability of domain stability should provide insight into the fundamental limits of ferroelectric memories and indicate prospective applications of ferroelectrics involving mechanical loads.

## Results

**Figure 1:**
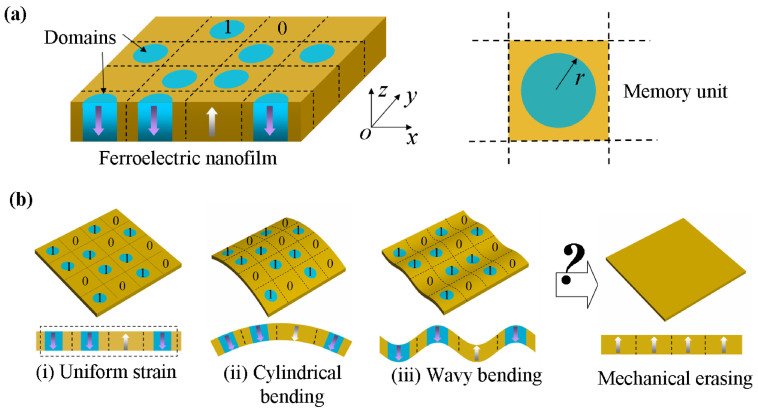
Schematic illustration of mechanical loads on stability of cylindrical domains in ferroelectric ultrathin films. (a) The model system. The left panel depicts the investigated subject, a ferroelectric nanofilm divided into rectangular memory units. Some of the memory units of the initially poled nanofilm are written with 180° cylindrical domains, to carry bit information. The right panel depicts the top view of a basic memory unit with a cylindrical domain of radius *r*. (b) Application of common mechanical loads to the nanofilm with written pattern to explore domain stability and the possibility of mechanical erasing. (i) Uniform strain, (ii) cylindrical bending, and (iii) wavy bending.

The basic idea of our investigation is illustrated in Figure 1. The model systems are (001) oriented PbTiO_3_ (PTO) ferroelectric ultrathin films divided into rectangular memory units. The conclusions of this investigation are believed to be applicable to other systems such as Pb(Zr*_x_*Ti_1-*x*_)O_3_ and BaTiO_3_ nanofilms, which have also been predicted to exhibit 180° domains. The PTO films are initially poled into single domain state, with all memory units initially carrying bit information “0”. Some memory units are then written with 180° inverted cylindrical domains to carry bit information “1”. The domain size is characterized by a lateral radius *r*. To see the effects of both strain and strain gradient on the domain stability, and to explore effective mechanical erasing mechanisms, we apply various mechanical loads to the nanofilm written with cylindrical domains, including (i) uniform strain, (ii) cylindrical bending and (iii) wavy bending, respectively. Note that cylindrical domain and control of its size can be achieved in SPM experiments by controlling strength and period of the SPM electric field^4^,^5^,^6^,^7^,^8^,^9^. Moreover, from an ideal point of view, the three mechanical loading conditions can be also realized in experiments by growing the PTO thin films on compliant substrates according to their elastic property. We therefore believe that the simulation results in this study can be verified in experiment, which we would nevertheless leave in a future wok.

### The critical size of stable cylindrical domain

**Figure 2:**
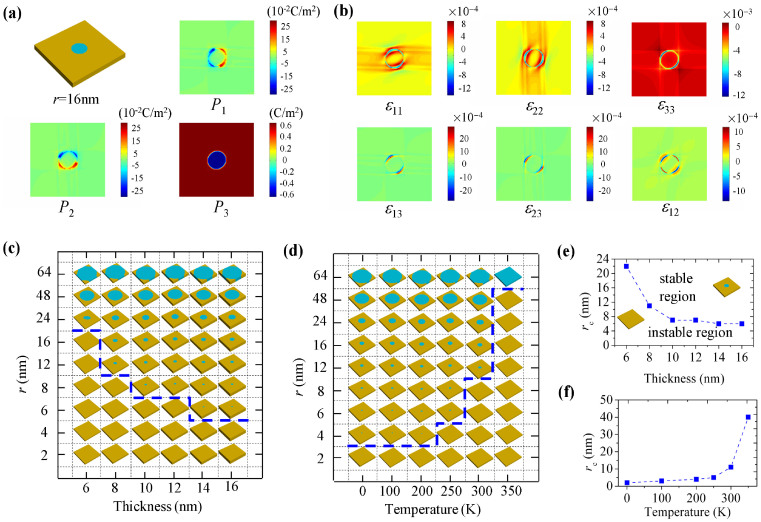
Phase diagram of equilibrium domain pattern. In-plane distributions of (a) polarization and (b) strain field at the top surface of a 128 nm × 128 nm × 8 nm simulation cell written with a cylindrical domain (*r* = 16 nm) at room temperature. Phase diagrams of equilibrium domain pattern in (c) 128 nm × 128 nm × *h* cells as a function of thickness *h* and domain size *r* at room temperature, and in (d) 128 nm × 128 nm × 8 nm cells as a function of temperature and domain size *r*. The cells are initially written with cylindrical domains with size *r* ranging from 1 nm to 64 nm. (e) and (f) the critical size of stable cylindrical domain as a function of thickness and temperature, respectively.

It is necessary to first gain an insight into the characteristics of 180° cylindrical domain in the nanofilm, which is important to understand its stability behavior. We simulate the equilibrium domain pattern of a 128 nm × 128 nm × 8 nm simulation cell at room temperature, which is free of external strain and initially written with a cylindrical domain of size *r* = 16 nm (see Supplementary Information on line for the generation of such a domain pattern). After polarization relaxation, the system is found to maintain the cylindrical domain pattern. Figure 2a and 2b depict the distributions of polarization and strain field viewed from the top surface (3D distributions are provided in Supplementary Figure S1 on line). The equilibrium domain pattern has nonzero in-plane polarization components *P*_1_ and *P*_2_ of about 0.03 C/m^2^ near the domain wall. Such a polarization rotation at 180° domain wall is in consistent with theoretical calculation^27^ and has been observed in experiment^28^. The domain wall is sharp with a maximum width of polarization rotation at the surface being about 2 nm, similar with the experimental measurement^28^. The strain components also have large magnitudes near the domain wall. Due to the large variation of *P*_3_ component across the domain wall, *ε*_33_, *ε*_13_ and *ε*_23_ have much larger magnitudes near the domain wall than the other strain components. Long-range elastic interaction between the cylindrical domains in adjacent cells is also seen from the distributions of normal strain components. It is worth noting that the distributions of polarization and strain field break the cylindrical symmetry around the central axis of the initial written pattern, due to polarization relaxation mediated by the anisotropic elastic effect. From these results, it can be seen that our simulation can well capture two important features of real 180° domain walls, i.e., polarization rotation near the domain wall and domain wall anisotropy. It is known that these two features of domain wall play important roles in its transport property^29^,^30^. In the following, we will show that they have great impact on the stability behavior of the nanodomain.

We continue to investigate the stability of cylindrical domains with considering the effects of film thickness and temperature. To achieve this, we write cylindrical domains with different radii into 128 nm × 128 nm × *h* simulation cells, which are free of external strain at different temperatures from *T* = 0 K to *T* = 350 K, with film thickness *h* ranging from 6 nm to 16 nm. The systems are relaxed to equilibrium to find whether the written domains can be maintained. The radius of the written cylindrical domains ranges from 1 nm to 64 nm. The simulated results are depicted in Figure 2c–f. For each investigated film thickness or temperature condition, it can be seen that there exists a critical size of cylindrical domain (denoted as *r*_c_), below which it is instable, shrinks and disappears in the simulation cell. Above the critical size, cylindrical domains with various sizes can maintain stable, indicating that they are actually metastable states. Obviously, this result reflects the multi-valleys morphology of the system's free energy. It is the barriers between the energy valleys that lead to a pinning force against domain wall shrinkage. We believe that this domain wall pinning effect is not an artificial effect but an intrinsic effect of real systems with periodic lattice potential. While it is difficult to be taken into account in analytic models, this intrinsic domain wall pinning effect is important for domain stability in defect-free systems.

Importantly, the stability of cylindrical domain is sensitive to film thickness. At *T* = 300 K, the critical size of cylindrical domain decreases from 22 nm to 6 nm when the film thickness increases from 6 nm to 16 nm (see Figure 2c and 2e). Considering the collective nature of domain, this result is easily understood as the domain has a larger volume in thicker film to withstand the devouring force from the surrounding domain. Apparently, such a dependence of domain stability on film thickness is against the well known Kittel's law^31^, which tells that the stable domain size increases as the film thickness increases. It is due to that our systems are under electrical short-circuit condition, thus the domain energy is mainly contributed by its bulk free energy rather than the depolarization energy. The latter is assumed to be dominant in the domain energy when deriving Kittel's law. A tradeoff between the increase of nanodomain density and the decrease of film thickness is clearly indicated by the simulated result. Similar behavior has been predicted in nanodomain formation under SPM tip by a phenomenological model considering the screening effect^32^.

For 128 nm × 128 nm × 8 nm simulation cells under different temperatures (see Figure 2d and 2f), it is found that at low temperatures the written cylindrical domain is rather stable with its critical size as small as 2 nm at *T* = 0 K. However, the critical size increases with the increase of temperature. At *T* = 350 K, *r*_c_ is found to be 48 nm. It is worth noting that there are strong nonlinear dependences of the cylindrical domain stability on both film thickness and temperature. Large margin of control on domain stability can be achieved at the high slope region of the *r*_c_-thickness or *r*_c_-temperature curves. On the other hand, if we were to make use of stable cylindrical domains, such nonlinear region should be kept away. Moreover, it should be pointed out that our predicted critical domain size is not necessary related with those obtained in SPM experiments^4^,^5^,^6^,^7^,^8^,^9^. This is largely due to the fact that the minimal domain size achieved in SPM experiments is limited by the radius curvature of SPM tip and is likely larger than our predicted critical domain size. Improved SPM technology and film grow method (to decrease the density of defects) may be necessary for inspection of the predicted “intrinsic” domain stability.

### Equilibrium domain patterns under different external biaxial strains

**Figure 3:**
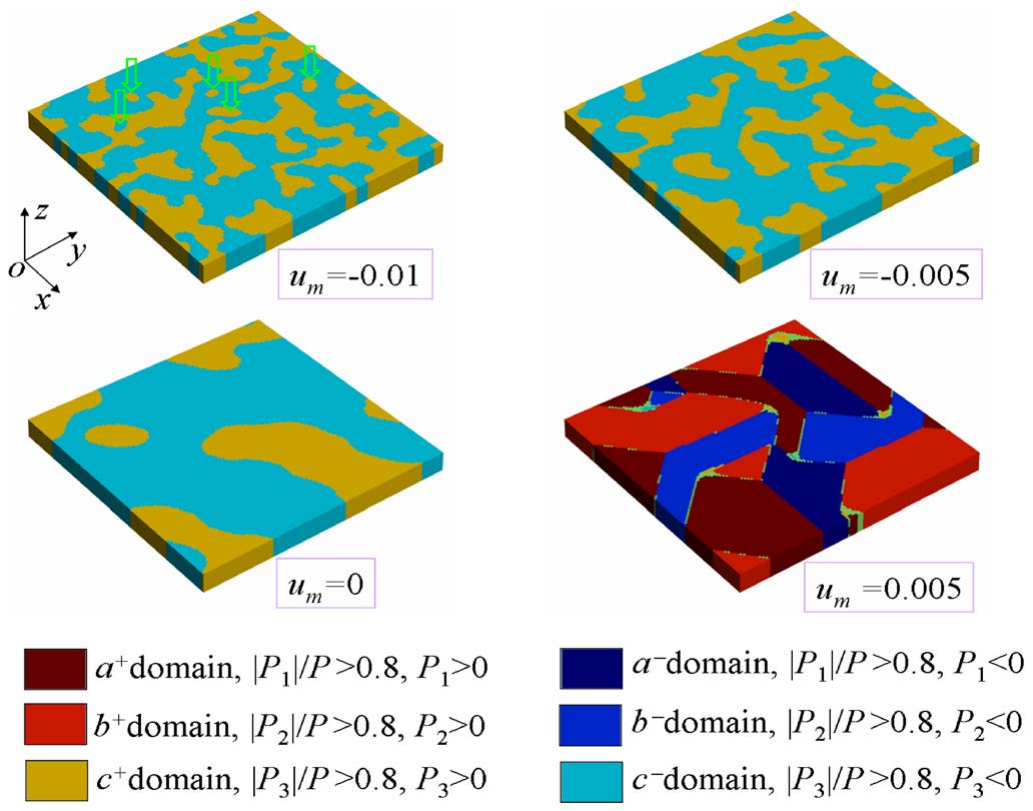
Equilibrium domain patterns under different external biaxial strains. Equilibrium domain patterns of a 128 nm × 128 nm × 8 nm simulation cell under different external biaxial strains, i.e., 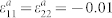, −0.005, 0 and 0.005. The cell is with an initial random polarization distribution. The green arrows indicate some small domains that are stable at 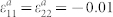.

After obtaining a basic insight into the stability of cylindrical domain as a function of film thickness and temperature, we focus on 8 nm-thick nanofilms at room temperature to explore the effects of mechanical loads. Mechanical loads effects on films with other thicknesses and/or at other temperatures can be qualitatively inferred. Before writing cylindrical domain patterns, in Figure 3, we first investigated the equilibrium domain patterns of a 128 nm × 128 nm × 8 nm simulation cell with initial random polarization distribution. The simulation cell is under different external biaxial strains, i.e., 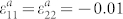, −0.005, 0 and 0.005. It can be seen that the domain morphology of the nanofilm is significantly dependent on the mechanical load. Specifically, it shows that in the first three loading conditions, the nanofilm is in *c*-domain (i.e., *P*_1_ = *P*_2_ = 0, *P*_3_ ≠ 0) structure, whereas in the last loading condition it is in *a/b*-domain (i.e., *P*_1_ ≠ 0, *P*_2_ = *P*_3_ = 0 or *P*_2_ ≠ 0, *P*_1_ = *P*_3_ = 0) structure. This is due to that *c*-domain structure and *a/b*-domain structure can respectively accommodate the compressive and tensile biaxial strains, leading to a decrease of elastic energy, as has been experimentally verified from the domain abundance in epitaxial PTO thin films^33^. Importantly, comparing the first three domain patterns, we notice that *c*-domain size is sensitive to the external strain. For example, as indicated by the green arrows, small domains are stabilized by compressive strain (−0.01). However, they disappear when the film is in a less compressive strain state (−0.005). More interestingly, the formed domain patterns are also different when the nanofilm is subjected to different strain gradient (see Supplementary Figure S2 on line). These results clearly indicate the sensitivity of 180° domain stability in nanofilms to external mechanical loads. In the following, we will make a comprehensive exploration on the 180° cylindrical domains stability as a function of different external mechanical loads.

### Control of domain stability by uniform strain

**Figure 4:**
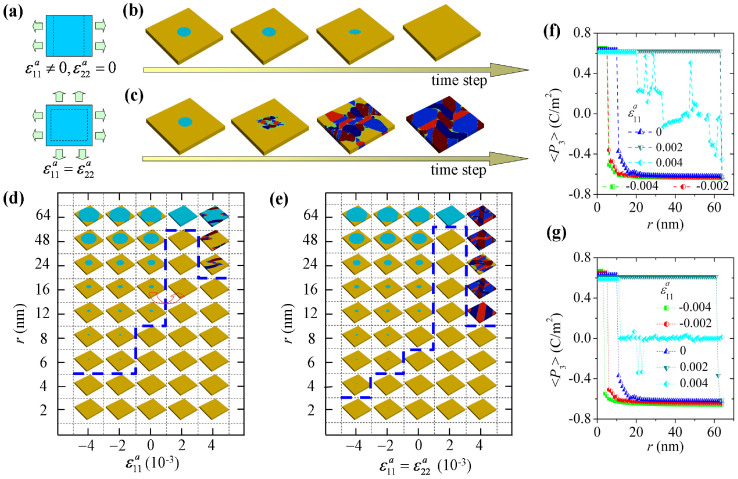
Control of domain stability by uniform strain. Control of domain stability by uniform strain on 128 nm × 128 nm × 8 nm simulation cells at room temperature. The cells are initially written with cylindrical domains with size *r* from 1 nm to 64 nm. (a) Schematics of a cell under biaxial strain (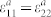) and uniaxial strain (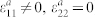). Domain evolution in a cell with cylindrical domain (*r* = 16 nm) under (b) 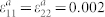 and (c) 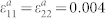. Phase diagrams of equilibrium domain pattern in cells under (d) uniaxial stain and (e) biaxial strain. (f) and (g) The average polarization of the equilibrium domain patterns in *z*-direction, i.e., <*P*_3_>, in the initial cylindrical domain region, for the two loading cases.

Cylindrical domains with different radii, i.e., ranging from 1 nm to 64 nm, are written into 128 nm × 128 nm × 8 nm simulation cells under different external mechanical loads to see how mechanical loads affect their stability. As shown in Figure 4a, for case of uniform external strain, we consider uniaxial strain condition (i.e., 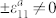 and 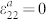) and biaxial strain condition (i.e., 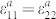). For both strain conditions, we generally found four typical evolution paths of the written domain pattern, dependent on the domain size and the strain condition. They are: (a) the cylindrical domain backswitches to a single domain state; (b) the cylindrical domain remains stable, probably with slight change in shape due to the anisotropic elastic effect; (c) the cylindrical domain expands to form a single domain state in cooperation with the adjacent cells; (d) the written domain pattern evolves into polydomain pattern with *a/b*-domains. In Figure 4b and 4c, we depict the domain evolution of the simulation cell with an initial 16 nm cylindrical domain at the strain conditions 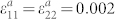 and 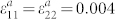, respectively. It shows that the cylindrical domain backswitches at 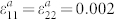. Meanwhile, it transforms into *a/b*-domains at 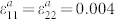. For this case, it is interesting to note that the *a*/*b*-domains nucleated at the domain wall, due to the polarization rotation at the domain wall as revealed in Figure 2. Moreover, the domain evolution is highly anisotropic, as a consequence of domain wall anisotropy and the favored orientation of *a*/*b*-domain walls along {110}, {100} and {010} planes of the prototypic cubic phase.

In order to summarize the regularity of uniform strain effect on cylindrical domain more clearly, in Figure 4d and 4e, we draw the “phase diagram” depicting the equilibrium domain pattern of the simulation cell as a function of written domain size and external strain for the two strain conditions, respectively. It is important to see that the stability of cylindrical domain strongly depends on external strain. Similar with the effects of film thickness and temperature, such dependences are highly nonlinear, indicating a large margin of controllability. Specifically, for the investigated range of strain and temperature, critical size of written cylindrical domain ranges from 6 nm to a value larger than 64 nm (at 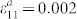 for both uniaxial and biaxial strain conditions). Note that instability of 64 nm cylindrical domain is tested in an extended simulation cell of size 256 nm × 256 nm × 8 nm to avoid domain expansion caused by the cooperation between adjacent cells. Otherwise, for 128 nm × 128 nm × 8 nm simulation cell as shown in the phase diagrams, the 64 nm cylindrical domain would evolve into a single domain state due to the cooperation between adjacent cells at 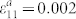 for both uniaxial and biaxial strain conditions. Furthermore, for the cases of largest strain, i.e., uniaxial strain (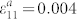, 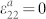) and biaxial strain (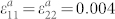), instead of remaining stable, the cylindrical domain would either backswitch or evolve into domain patterns with *a/b*-domains. Comparing the uniaxial and biaxial strain cases, it can be also seen that the abundance of *a/b*-domains in those patterns is much larger in biaxial strain condition.

In Figure 4f and 4g, we calculated the average polarization in *z*-direction over the initial cylindrical region of the written domain, denoted as <*P*_3_>. The critical size of stable cylindrical domain and its dependence on external strain are clearly seen, as indicated by the transformation from instable state to stable state with a changing sign of <*P*_3_>. It can be also inferred that those stable cylindrical domains probably have slight change in shape, particularly when the domain size is small, according to the round corner section right after transformation point of the <*P*_3_> curves. Moreover, significant rumplings are observed in the <*P*_3_> curves for the cases of largest strain, due to the fact that *a/b*-domains are induced and their morphologies are sensitive to the size of the initial cylindrical domain.

Note that Figure 4d and 4e present the equilibrium phase diagrams of cylindrical domain as a function of domain size and external strain. From these phase diagrams, we can readily infer the size range of stable cylindrical domain in an initially poled nanofilm for memory applications. Importantly, this size range can be effectively controlled by external strain. Through applying compressive external strain, the stability of cylindrical domain would be significantly enhanced and thus increases the storage density and data stability. Even more important indication from these phase diagrams is that we can erase the domain by changing the strain state. As indicated by the arrow in Figure 4d, if we write relatively small domain at compressive strain condition, by changing the strain condition to be less compressive, we can erase the domain. Interestingly, further simulation shows that the induced *a/b*-domains by uniaxial strain (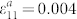, 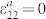) are instable if the strain is removed, leading to a single (poly) *c*-domain state when the initial cylindrical domain size is small (large). This indicates that mechanical erasing can be achieved even though *a/b*-domains were induced. Nevertheless, this seems only possible when the abundance of induced *a/b*-domains is relatively small. For those patterns with a large abundance of induced *a/b*-domains at biaxial strain condition (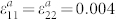), most of them would relax to poly *c*-domain state or maintain stable, leading to an unsuccessful erasing. Typical evolution paths of domain patterns with induced *a*/*b*-domains after the strain load is off can be found in Figure S3 in Supplementary Information on line.

### Control of domain stability by cylindrical bending

Besides uniform external strain, a ferroelectric nanofilm can be subjected to mechanical bending loads, which impose strain gradient and/or strain to the nanofilm. It is of practical significance to explore an effective mechanical way to erase nanodomains. The investigation of mechanical bending effects on the domain stability of ferroelectric nanofilm is also important for applications of flexible ferroelectric devices. Therefore, in the following we will inspect another two mechanical loading conditions, i.e., cylindrical bending condition and wavy bending condition. Note that the existence of strain gradient *ε_jk,l_* may lead to a flexoelectric field, i.e., 
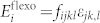
, with *f_ijkl_* being the flexocoupling coefficients^25^. This flexoelectric field brings a mixed mechanical and electrical feature to the strain gradient effect. Currently, flexoelectricity is still a puzzled phenomenon in literature despite its growing relevance. Particularly, for flexocoupling coefficients, there are orders of magnitude in discrepancy between the theoretical estimations and experimental measurements^25^. Considering this uncertainty of the flexocoupling coefficients, and to see the strain gradient effect on domain stability more clearly, in the following we take values of the flexocoupling coefficients in between those of theoretical estimations and experimental measurements, and conduct simulations with artificially switching on/off the flexoelectric field.

**Figure 5:**
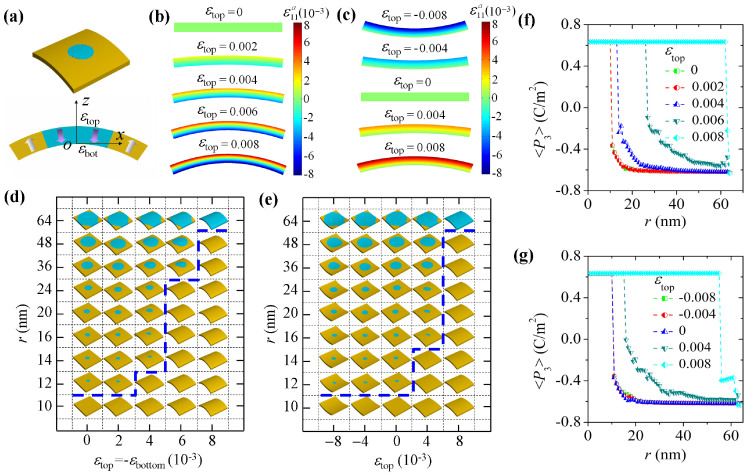
Control of domain stability by cylindrical bending. Control of domain stability by cylindrical bending on 128 nm × 128 nm × 8 nm simulation cells at room temperature. The cells are initially written with cylindrical domains with size *r* from 1 nm to 64 nm. (a) Schematics of a cell under cylindrical bending, i.e., 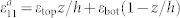. Distribution of 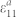 as a function of *ε*_top_ in the *x*-*z* plane of a cell under (b) pure bending (*ε*_top_ = −*ε*_bot_) and (c) mixed bending-strain (*ε*_top_ ≠ 0, *ε*_bot_ = 0). Phase diagrams of equilibrium domain pattern in cells under (d) pure bending and (e) mixed bending strain conditions. (f) and (g) The average polarization of the equilibrium domain patterns in *z*-direction, i.e., <*P*_3_>, in the initial cylindrical domain region, for the two bending cases.

For the cylindrical bending condition, we mean that the nanofilm is under an external bending strain in form of 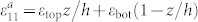, where *ε*_top_ and *ε*_bot_ are the magnitudes of 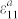 at the top and bottom surfaces of the film, respectively, and coordinate *z* is measured with respect to the bottom surface of the film. This bending strain would cause a cylindrical-shape bending of the nanofilm along the *x*-direction as shown in Figure 5a. Typically, we consider two loading cases, i.e., the pure bending case with zero membrane strain, i.e., 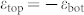, and mixed bending-strain case with nonzero membrane strain, i.e., *ε*_top_ ≠ 0 and *ε*_bot_ = 0. The distribution of 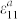 as a function of *ε*_top_ in the *x*-*z* plane of an 8 nm-thick nanofilm for the two loading cases is depicted in Figure 5b and 5c, respectively.

Now we write cylindrical domains with different radii, i.e., ranging from 1 nm to 64 nm, into 128 nm × 128 nm × 8 nm simulation cells under different cylindrical bending strains at room temperature. The corresponding phase diagrams depicting the equilibrium domain pattern as a function of domain size and bending strain are shown in Figure 5d and 5e, for the pure bending case and mixed bending-strain case respectively. Note that the flexoelectric field has been neglected to see the pure mechanical effect. From the phase diagrams, it can be seen that cylindrical bending can also effectively control the stability of cylindrical domain. Firstly, it shows that strain gradient decreases the stability of cylindrical domain, similar with the effect of tensile strain. Nevertheless, strain gradient can largely depress the formation of *a/b*-domains. Moreover, dependence of the domain stability on cylindrical bending also exhibits a strong nonlinear feature. For the pure bending case (Figure 5d), it shows that the critical size of stable cylindrical domain remains almost the same when the bending strain is small, i.e., *ε*_top_ = −*ε*_bot_ < 0.004. However, as the bending strain further increases, the critical size of stable cylindrical domain rapidly increases to ~30 nm at *ε*_top_ = −*ε*_bot_ = 0.006, and exceeds 64 nm at *ε*_top_ = −*ε*_bot_ = 0.008 (To see the instability of 64 nm cylindrical domain, the simulation cell size is extended to be 256 nm × 256 nm × 8 nm). This nonlinear feature is even more significant in the mixed bending-strain condition (Figure 5e), due to the superposition of strain gradient effect and membrane strain effect. For this loading case, the membrane strain is compressive when *ε*_top_ < 0, which cancels the effect of strain gradient, leading to small change of domain stability. Meanwhile, the membrane strain becomes tensile when *ε*_top_ > 0, which has similar effect of strain gradient, and leads to large change of domain stability. This indicates a larger margin of controllability on the cylindrical domain stability of this type of mechanical loading.

In Figure 5f and 5g, corresponding to the two cylindrical bending cases, we also calculated the average polarization in *z*-direction over the initial cylindrical region of the written domain. The critical size of stable cylindrical domain and its dependence on cylindrical bending strain are clearly seen, as indicated by the transformation from instable state to stable state with a changing sign of <*P*_3_>. It should be also noted that the <*P*_3_> of single domain state almost remains the same despite the large change of bending condition. Moreover, compared with the cases of uniform strain loading conditions as shown in Figure 4, the stable cylindrical domains change their shape more significantly in cylindrical bending conditions, as indicated by the round corner section right after transformation point of the <*P*_3_> curves.

### Control of domain stability by wavy bending

**Figure 6:**
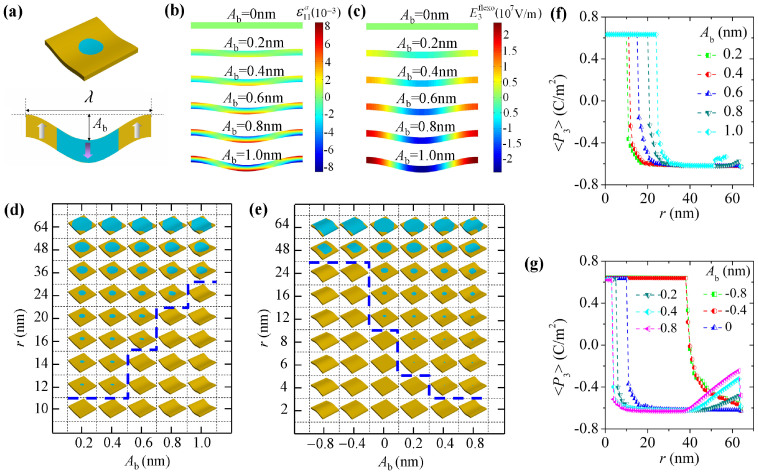
Control of domain stability by wavy bending. Control of domain stability by wavy bending on 128 nm × 128 nm × 8 nm simulation cells at room temperature. The cells are initially written with cylindrical domains with size *r* from 1 nm to 64 nm. (a) Schematics of a cell under wavy bending, i.e., 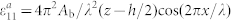, with *λ* = 128 nm. Distribution of (b) 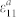 and (c) flexoelectric field 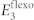 as a function of *A*_b_ in the *x*-*z* plane of a cell under wavy bending. Phase diagrams of equilibrium domain pattern in cells under wavy bending with flexoelectric field (d) switched off and (e) on. (f) and (g) The average polarization of the equilibrium domain patterns in *z*-direction, i.e., <*P*_3_>, in the initial cylindrical domain region, for the two bending cases.

The investigated mechanical loading conditions so far are with uniform strain or uniform strain gradient. It has been recently demonstrated that a wavy bending configuration of ferroelectric nanofilm can be achieved by integrating it on prestrained elastomeric supports, which is important for developing flexible ferroelectric devices^15^,^16^ Considering the fact that the strain gradient of wavy bending is not uniform, an exploration on its effect is not trivial to the cases of uniform strain and uniform strain gradient. For a nanofilm with a wavy bending along *x*-direction (Figure 6a), its strain state can be approximately described by a cosine form strain, i.e., 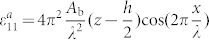, where *A*_b_ is wave amplitude, characterizing the largest defection of the nanofilm from its neutral plane *z* = *h*/2, and *λ* is the wave length along the *x*-direction. Note that for simplicity we fix the neutral plane at middle plane of the film to only consider the case of zero membrane strain.

To see how wavy bending affects the stability of 180° cylindrical domain, simulations are conducted on 128 nm × 128 nm × 8 nm simulation cells under different wavy bending strains (*λ* = 128 nm) at room temperature. The simulation cells are initially written into cylindrical domains, with their radii ranging from 1 nm to 64 nm. Figure 6b depicts the distributions of 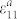 as a function of *A*_b_ in the *x-z* plane of the simulation cell under a wavy bending with *λ* = 128 nm. Here we also investigate the effect of wavy bending in the presence of a flexoelectric field, 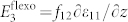, with *f*_12_ = 10 V. The distribution of 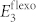 as a function of *A*_b_ in the *x-z* plane of the simulation cell is depicted in Figure 6c. Thus in Figure 6d and 6e, we respectively calculate the phase diagram of equilibrium domain patterns in the simulation cells for two wavy bending cases, i.e., with zero and nonzero flexoelectric field, respectively. The average polarization in *z*-direction, i.e., <*P*_3_>, over the initial cylindrical region of the written domain, are also calculated and depicted in Figure 6f and 6g, corresponding to the two bending cases.

Firstly, it can be seen that wavy bending has significant impact on the stability of cylindrical domains. As shown in Figure 6d, where the flexoelectric field is switched off, the critical size of stable cylindrical domain changes from 11 nm to 30 nm when the bending amplitude *A*_b_ increases from 0.2 nm to 1.0 nm. Note that although the maximum strain of a wavy bending with *A*_b_ = 10 nm is about 0.01 according to 
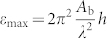
, the controllability of investigated wavy bending is less significant than the uniform in-plane stains (Figure 4) and cylindrical bending (Figure 5). This is due to the fact that, for a large size of domain, its stability is more likely to be affected by the overall strain and strain gradient state. Therefore, if the flexoelectric field is negligible, for the margin of controllability on cylindrical domain stability, we have uniform in-plane strain > cylindrical bending (with zero membrane strain) > wavy bending.

Nevertheless, significant difference can be found in the controllability on domain stability of the bending loads, if a flexoelectric effect is not trivial. Specifically, flexoelectric effect imposes an electric field proportional to the strain gradient, and changes sign as direction of the strain gradient changes. As a consequence, both enhancement and depression of the stability of the 180° cylindrical domain are possible, dependent on the direction of the domain polarization and the flexoelectric field. This feature is clearly seen in Figure 6e, where a flexoelectric field is included. Specifically, the critical size of stable cylindrical domain is tuned from 3 nm to 48 nm, dependent on the bending direction. Note that upper limit of 48 nm exists in the upper bending case due to the cosine form of the strain gradient. A much larger critical domain size can be reached by increasing the wave length or in case of cylindrical bending. Note also that the maximum flexoelectric field is about 2.5 × 10^7^ V/m in our investigation (Figure 6b). In normal ferroelectrics, polarization switching occurs at much lower field strengths than that predicted by Landau-type phenomenology. The flexoelectric field needed to significantly affect the stability of cylindrical domains might be much less than our prediction. Due to the modulated flexoelectric field, large change in domain shape not only happens at region near domain instability but also happens when the domain size is comparable to the wave length, as indicated by the <*P*_3_> curves as shown in Figure 6g.

### Mechanical erasing of the information in nano-ferroelectric memories

**Figure 7:**
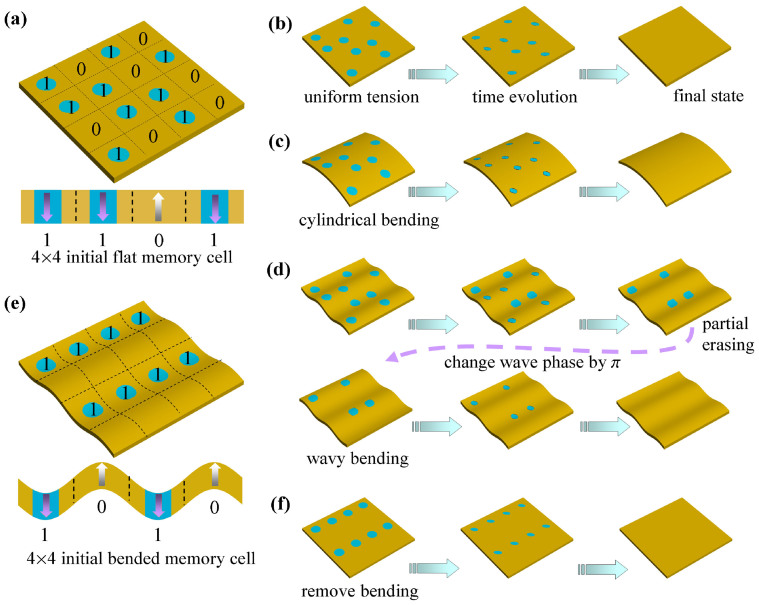
Mechanical erasing of the information in nano-ferroelectric memories. Mechanical erasing of the information in 4 × 4 bits memory systems. (a) A 256 nm × 256 nm × 8 nm memory system written with 4 × 4 bits of information carried by cylindrical domains of *r* = 16 nm. Total erasing of the information of this system is demonstrated by applying (b) uniform biaxial strain (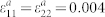) or (c) cylindrical bending (*ε*_top_ = −*ε*_bot_ = 0.006). (d) Making use of nontrivial flexoelectric effect, partial erasing can be achieved by applying wavy bending (*A*_b_ = 1.0 nm and *λ* = 128 nm) to the system. By changing the bending wave phase by π, the remaining information is also erased. (e) A 256 nm × 256 nm × 6 nm memory system written stable information at the valleys under wavy bending (*A*_b_ = 0.2 nm and *λ* = 128 nm). (f) The information is erased after removing the wavy bending.

Finally, we would like to conduct simulations on multi-bits memory system with 4 × 4 memory units at room temperature, to clearly show how to erase the information by exerting or removing mechanical loads. In Figure 7a, a 256 nm × 256 nm × 8 nm memory system at free of external strain condition is initially written with 4 × 4 bits of information, with the size of written cylindrical domains being 16 nm. The information is found stable at such a strain state, in consistent with the phase diagrams in Figures 3,4,5,6. Then we apply external strain to the memory system. Figure 7b shows that evolution of the information as the film is under uniform biaxial strain, 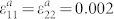. It can be seen that the information is totally erased, due to the instability of 16 nm cylindrical domain at this loading condition. The erasing of the information can be also done by applying cylindrical bending (*ε*_top_ = −*ε*_bot_ = 0.006) to the memory system (Figure 7c). Interestingly, making use of the flexoelectric effect, we can also partially erase the information by applying a wavy bending (*A*_b_ = 1.0 nm and *λ* = 128 nm) to the system (Figure 7d). Moreover, the remaining information can be also erased by changing the phase of bending wavy, e.g., by π. Another mechanical erasing example exploiting the flexoelectric effect is illustrated in Figure 7e and 7f. In Figure 7e, it shows that a 256 nm × 256 nm × 6 nm memory system under wavy bending (*A*_b_ = 0.2 nm and *λ* = 128 nm) can maintain 16 nm cylindrical domains at the valleys, due to enhance of domain stability by the flexoelectric effect. After removing the wavy bending, as 16 nm cylindrical domain is no longer stable, the information is successfully erased (Figure 7f). These results clearly demonstrate the possibility of erasing the information by mechanical loads.

## Discussion

We have conducted a systematical investigation to explore the effects of macroscopic mechanical loads on 180° cylindrical domain patterns in ferroelectric ultrathin films, and the possibility of mechanical erasing the ferroelectric domains. Dependence of cylindrical domain stability on domain size, film thickness and temperature has been revealed. Subsequently, control on the cylindrical domain stability of different mechanical loads, including uniform strain, cylindrical bending, and wavy bending, has been comprehensively investigated and compared. Our results show that both strain and strain gradient have great impact on the stability of 180° cylindrical domain, with its critical size sensitive to strain state of the nanofilm. Particularly, we found that if the flexoelectric field is neglected, the cylindrical domain stability is most sensitive to uniform in-plane strain, and least sensitive to wavy bending. Meanwhile, if a flexoelectric effect is not trivial, significant difference can be found in the controllability on domain stability of the bending loads. All these results indicate erasing 180° cylindrical domain can be achieved by changing the strain state of the nanofilm. According to the calculated phase diagrams depicting the cylindrical domain stability as a function of mechanical loads and domain size, we successfully simulated possible mechanical erasing processes of a memory device storing 4 × 4 bits information. We believe our finding is instructive to prospective device applications of ferroelectric ultrathin films, such as flexible memory devices and other micro-electromechanical systems based on nanodomains.

Here we would like to make a further discussion about the indications of large controllability of mechanical loads on cylindrical domain stability. Besides utilizing this controllability to increase storage density of cylindrical domains and to realize mechanical erasing of the nanodomains, some interesting points can be further drawn. Obviously, the coercive field of domain switch, and thus the operating voltage and speed, should be also strong functions of mechanical loads. In general, there is always a tradeoff in the pursuit of high density, low coercive field/operating voltage, and high operating speed. By exploring the dependence of related factors on mechanical loads, an optimized point at which the memory device is of high-density, high-speed and low-power consumption can be reached. An even more clever strategy is to maintain the nanodomain of memory device at a mechanical strain state and to process the domain at another mechanical strain state. Moreover, for memory devices under the wavy bending, our simulation indicates that the domain stability is spatial dependent. Making use of this property, we can realize addressable erasing of part of the information if we can precisely control the wavy pattern (e.g., wave length and wave phase). Moreover, information can be stored at different regions according to their operating condition (for example, information that needs frequent operation can be stored at less stable region). This would be of practical importance for applications. All of these, although there is still a long path to go through, indeed present us wonderful possibility in memory applications involving mechanical loads.

## Methods

Typically, the PTO nanofilms are modeled as infinite along the in-plane *x*- and *y*- directions. We mainly restrict ourselves to simulation cells containing one inverted cylindrical domain at the center region (Figure 1), and apply periodic conditions in *x*- and *y*-directions. Simulation cells with 4 × 4 memory units to mimic multi-bits memory devices will be also investigated to show how to erase the information by adjusting mechanical loads. The nanofilms are assumed to be under in-plane strain constraint with free top-bottom surfaces and under electrical short-circuit condition^18^. Periodic conditions are applied in the in-plane directions. Meshing grids of *n_x_*Δ*l* × *n_y_*Δ*l* × *n_z_*Δ*l* are used to simulate different simulation cells, with scale Δ*l* equal to 1 nm.

The domain structure is solved by a generally accepted and powerful theory for simulating domain structures of ferroelectrics, i.e., the phase field model based on the Landau-type formalism, in which we also carefully takes into account the effects of inhomogeneous mechanical field, electric filed and surfaces. Such approach has been widely used to simulate ferroelectric domain structure, and represents well the experimental results of ferroelectric domain structures in a wide range of materials and conditions^33^,^34^,^35^,^36^. Specifically, in our phase field model, evolution of the polarization field is described by the Time-Dependent Ginzburg-Landau (TDGL) equations, i.e., ∂*P_i_*/∂*t* = −*MδF*/*δP_i_*, where *F* is the free energy of the system, *P_i_* are the polarization components, *t* is time, and *M* is the kinetic coefficient related to the domain wall mobility. The evolution of elastic and electric fields are treated in an “adiabatic” way. At each time step of polarization evolution, these fields are calculated according to the mechanical and electrostatic equilibrium equations, using a fast Fourier transformation (FFT) technique based on Khachaturyan's microscopic elasticity theory^37^ and Stroh formulism of anisotropic elasticity^38^. In literature, based on this approach, the domain structures in epitaxial ferroelectric thin films have been successfully simulated^17^,^18^. To guarantee the convergence of domain structure to equilibrium, the simulation time is set to be sufficient long (up to 10^6^ time steps). The material parameters in calculations are listed Table 1. For PTO, a commonly used six-order Landau-potential is adopted in this study. The potential coefficients were determined by fitting the experimental results thus the potential captures well the phase transition behavior of PTO^39^. Values of the expansion coefficients of the Landau potential, elastic and electrostrictive coefficients are taken from Ref. 23. Due to the lack of anisotropy gradient coefficients, they are taken as isotropic similar with Ref. 17. Note that a larger set of gradient coefficients generally leads to a larger critical size of cylindrical domain, in a similar way of temperature. The extrapolation length is from Ref. 40, and the background dielectric constant is chosen according to Ref. 41 and 42. The magnitude of flexocoupling coefficient *f*_12_ (~10 V) is a roughly estimated to lie between the theoretical estimations and experiment measurements, due to the uncertainty of this parameter of the material^25^. Detailed description of phase field method, details of “writing” cylindrical domain patterns, 3D polarization and strain distributions of cylindrical domain pattern, and strain gradient effect on formation of domain structure of nanofilm are provided in the Supplementary Information on line.

## Author Contributions

Y.Z. initiated and performed this work and manuscript. W.J.C. and Y.Z. conceived and designed the basic idea, structures and simulations. X.F. and B.W. suggested the principle idea. W.J.C. performed the simulations. Y.Z., W.J.C., X.F., D.C.M. and Y.W. analyzed the results of simulations. Y.Z., W.J.C. and B.W. co-wrote the manuscript. All authors contributed to discussion and reviewed the manuscript.

## Supplementary Material

Supplementary Information Supplementary Information

## Figures and Tables

**Table 1:** Values of parameter used in the phase field simulations (SI units and *T* in K)

Parameter	Value	Unit
*a*_1_	3.85(*T*−752) × 10^5^	C^−2^m^2^N
*a*_11_	−7.3 × 10^7^	C^−4^m^6^N
*a*_12_	7.5 × 10^8^	C^−4^m^6^N
*a*_111_	2.6 × 10^8^	C^−6^m^10^N
*a*_112_	6.1 × 10^8^	C^−6^m^10^N
*a*_123_	−3.7 × 10^9^	C^−6^m^10^N
*c*_11_	1.746 × 10^11^	Nm^−2^
*c*_12_	0.7937 × 10^11^	Nm^−2^
*c*_44_	1.1111 × 10^11^	Nm^−2^
*Q*_11_	0.089	C^−2^m^4^
*Q*_12_	−0.026	C^−2^m^4^
*Q*_44_	0.0675	C^−2^m^4^
*G*_110_	1.73 × 10^−10^	m^4^NC^−2^
*G*_11_	*G*_110_	m^4^NC^−2^
*G*_12_	0	m^4^NC^−2^
*G*_44_	0.5*G*_110_	m^4^NC^−2^
*G*_44_′	0.5*G*_110_	m^4^NC^−2^
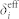	5 × 10^−9^	m
*ε_b_*	4.425 × 10^−10^	Fm^−1^
*f*_12_	10	V

*a*_1_, *a*_11_, *a*_12_, *a*_111_, *a*_112_, *a*_123_ are the coefficients of Landau potential, *c_ij_* are the elastic coefficients, *Q_ij_* are the electrostrictive coefficients, *G*_11_, *G*_12_, *G*_44_, *G*_44_′ are gradient coefficients, 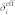 are the extrapolation lengths, *ε_b_* is the background dielectric constant, and *f*_12_ is the flexocoupling coefficient.
