# Supplementary material for: Effect of Mechanical Loads on Stability of Nanodomains in Ferroelectric Ultrathin Films: Towards Flexible Erasing of the Non-Volatile Memories
Source: Sci Rep. 2014 Jun 18;4:5339. doi: 10.1038/srep05339 (PMC4061556; doi:10.1038/srep05339)
Supplement: Supplementary Information [file srep05339-s1.pdf]

Supplementary Information for

Effect of Mechanical Loads on Stability of Nanodomains in Ferroelectric Ultrathin Films: Towards Flexible Erasing of the Non-Volatile Memories?

W. J. Chen[1,2], Yue Zheng[1,2], W. M. Xiong[1,2], Xue Feng[3], Biao Wang[1,4] and Ying Wang[1,2]

[1]State Key Laboratory of Optoelectronic Materials and Technologies, School of Physics and Engineering, Sun Yat-sen University, Guangzhou 510275, China.
[2]Micro&Nano Physics and Mechanics Research Laboratory, School of Physics and Engineering, Sun Yat-sen University, Guangzhou 510275, China.
[3]AML, Department of Engineering Mechanics, Tsinghua University, Beijing 100084, China.
[4]Sino-French Institute of Nuclear Engineering and Technology, Zhuhai Campus, Sun Yat-sen University, Zhuhai 519082, China.

1. The phase-field model

In phase-field models, the domain structure of a ferroelectric is described by the order parameter, i.e., the spontaneous polarization $\boldsymbol{P} = \left(P_1, P_2, P_3\right)$. The electric displacement field is expressed in terms of the electric field, linear-part induced polarization and nonlinear-part spontaneous polarization as,

$$\boldsymbol{D} = \boldsymbol{\varepsilon}_0 \boldsymbol{E} + \boldsymbol{P}^{\mathrm{T}} = \boldsymbol{\varepsilon}_0 \boldsymbol{E} + \boldsymbol{\chi}_{\mathrm{b}} \boldsymbol{E} + \boldsymbol{P} = \boldsymbol{\varepsilon}_{\mathrm{b}} \boldsymbol{E} + \boldsymbol{P} \tag{1}$$

where $\boldsymbol{E} = -\nabla \varphi$ is the electric field, φ the electric potential, $\boldsymbol{\chi}_{\mathrm{b}}$ the background susceptibility tensor, $\boldsymbol{\varepsilon}_0$ the vacuum permittivity and $\boldsymbol{\varepsilon}_{\mathrm{b}} \equiv \boldsymbol{\varepsilon}_0 + \boldsymbol{\chi}_{\mathrm{b}}$ the dielectric constant tensor of the background materials. Since the background material is in cubic paraelectric phase, the background dielectric constants in three axis directions are the same, i.e., $\varepsilon_b = \varepsilon_{11b} = \varepsilon_{22b} = \varepsilon_{33b}$.

The evolution of the polarization field toward its equilibrium distribution is driven by the decrease of the free energy of the system, which is phenomenologically described by the Time Dependent Ginzburg–Landau (TDGL) equations, i.e.,

$$\frac{\partial P_i}{\partial t} = -M \frac{\delta F}{\delta P_i}, \ (i = 1, 2, 3) \tag{2}$$

where M is the kinetic coefficient related to the domain wall mobility and t is time.

Based on the Landau-Ginzburg-Devonshire (LGD) phenomenological theory, the free energy of the system is expressed as a functional of order parameter field and applied fields. For the ferroelectric nanofilm considered here, taking into account effects of the mechanical strain, electric field and surface, spatial polarization variation, the total free energy could be written as the sum of the Landau free energy F_{Land}, elastic energy F_{elas}, gradient energy F_{grad}, electrostatic energy F_{elec} and surface

energy F_{surf}, that is,

$$\begin{aligned} F &= F_{\text{Land}} + F_{\text{elas}} + F_{\text{grad}} + F_{\text{elec}} + F_{\text{surf}} \\ &= \int_V \left(f_{\text{Land}} + f_{\text{elas}} + f_{\text{grad}} + f_{\text{elec}} \right) dV + \int_S f_{\text{surf}} \, dS \end{aligned} \tag{3}$$

where f_{Land}, f_{elas} f_{grad}, f_{elec} and f_{surf} are the corresponding free energy densities, V and S the volume and surface of the nanofilm.

For perovskite ferroelectrics of cubic symmetry in paraelectric phase, the Landau free energy density f_{Land} can be generally expressed up to a six-order polynomial expansion for a zero stress as[1-2],

$$\begin{aligned} f_{\text{Land}} &= a_1 \sum_i P_i^2 + a_{11} \sum_i P_i^4 + a_{12} \sum_{i>j} P_i^2 P_j^2 + a_{111} \sum_i P_i^6 \\ &\quad + a_{112} \sum_{i>j} \left(P_i^4 P_j^2 + P_j^4 P_i^2 \right) + a_{123} \prod_i P_i^2 \end{aligned} \tag{4}$$

where a_i, a_{ij}, and a_{ijk} are dielectric stiffness and higher order coefficients fitted to bulk properties and P_i is the ith component of polarization.

Under the condition of applied mechanical strain, the mechanical strain field (applied and internal) and its coupling with polarization contribute to the elastic energy density, which is described by,

$$f_{\text{elas}} = \tfrac{1}{2} c_{ijkl} e_{ij} e_{kl} = \tfrac{1}{2} c_{ijkl} (\varepsilon_{ij} - \varepsilon_{ij}^0)(\varepsilon_{kl} - \varepsilon_{kl}^0) \tag{5}$$

where c_{ijkl} are the fourth-rank elastic stiffness coefficients, e_{ij} are the elastic strain components, ε_{ij}^0 are the eigenstrain components, and ε_{ij} are the total strain components, which must be compatible and are related to the displacement as $\varepsilon_{ij} = \tfrac{1}{2}(u_{i,j} + u_{j,i})$. For perovskite ferroelectrics with cubic symmetry in paraelectric phase, the eigenstrain components are given by $\varepsilon_{ij}^0 = Q_{ijkl} P_k P_l$, with Q_{ijkl} being the fourth-rank electrostrictive coefficients.

The spatial polarization variation contributes a gradient energy to the total free

energy. To the lowest order of Taylor expansion, the gradient energy density takes the form as $f_{\text{grad}} = \frac{1}{2} G_{ijkl} P_{i,j} P_{k,l}$, with G_{ijkl} being the fourth-rank gradient energy coefficients. Due to truncation at the surface of the nanofilm, the spontaneous polarization is inhomogeneous across the out-of-plane direction. Thus an additional surface energy is necessary to describe this intrinsic effect. Using the so-called extrapolation length[3], the surface energy density of the ferroelectric nanoplatelet can be approximately given by $f_{\text{surf}} = \frac{1}{2} D_i^S P_i^2 / \delta_i^{\text{eff}}$, where δ_i^{eff} are extrapolation length and D_i^S the material coefficients related to the gradient energy coefficients and the surface orientation. According to the previous works[4-6], the electric energy density of a given polarization distribution is written as $f_{\text{elec}} = -P_i E_i - \frac{1}{2} \varepsilon_b E_i E_i$ .In the absence of external electric field, the total electric field is equal to depolarization field induced by spatial polarization variation and incomplete screening of the polarization charges at truncated surfaces. Moreover, if the flexoelectric effect is considered[7], the presence of strain gradient will contribute to a flexoelectric field, i.e., $E_i^{\text{flexo}} = f_{ijkl} \varepsilon_{jk,l}$, with f_{ijkl} being the so-called flexocoupling coefficients.

The phase-field simulations are conducted by numerically solving the TDGL equations (2) together with the proper solution of the inhomogeneous mechanical and electric fields. The TDGL equations are solved are solved using the finite difference method. Meanwhile, the mechanical and electric fields are solved using a fast Fourier transformation (FFT) technique based on the Khachaturyan's microscopic elastic theory[8] and Stroh formalism of anisotropic elasticity[9] (see the following sections). The nanofilms are assumed to be under strain constraint and electrical short-circuit

conditions. Periodic conditions are applied in the in-plane directions. Meshing grids of $128\Delta l \times 128\Delta l \times n_z \Delta l$ and $256\Delta l \times 256\Delta l \times n_z \Delta l$ are used to simulate different memory cells, with scale Δl equal to 1nm. To guarantee the convergence of domain structure to equilibrium, the simulation time is set to be sufficient long (up to 10^6). The time step is chosen to be $\Delta t = 0.01 a_0 M$, where $a_0 = \left|a_1\right|_{T=300K}$. Values of the expansion coefficients of the Landau-potential, electrostrictive coefficients, elastic properties in calculations are listed Table S1. For $PbTiO_3$, a commonly used six-order Landau-potential is adopted in this study.

2. Elastic field of a nanofilm

In the following, we consider the elastic field of a nanofilm subjected to external strain constraint in the film plane, i.e., $\varepsilon^a_{\alpha\beta}$, with $\alpha, \beta = 1, 2$. The two surfaces of the nanofilm, i.e., z=0 and z=h, are assumed to be free of traction. To take into account the bending of nanofilm, $\varepsilon^a_{\alpha\beta}$ are allowed to vary along the z direction. In terms of displacement, the mechanical equilibrium equation and boundary conditions are given by

$$c_{ijkl} u_{k,lj} = c_{ijkl} \varepsilon^0_{kl,j} = \sigma^0_{ij,j}(\vec{r}) \tag{5a}$$

$$c_{i3kl} u_{k,l}\Big|_{z=0,h} = c_{i3kl} \varepsilon^0_{kl}\Big|_{z=0,h} \tag{5b}$$

$$u_i\big|_{x=0} = u_i\big|_{x=lx}, u_i\big|_{x=0} = u_i\big|_{x=ly} \tag{5c}$$

where periodic conditions have been applied along the in-plane x and y directions, with l_x and l_y being the lengths of the simulation cell along the x and y directions, and $\sigma^0_{ij,j}(\vec{r}) = c_{ijkl} \varepsilon^0_{kl,j}(\vec{r})$.

We take three steps to solve this elastic field problem. Following the microscopic elastic theory of Khachaturyan[8,10], we first solve Equation (5a) with periodic conditions in all the three dimensions. The solution is labeled as $u_i^A(\vec{r})$. This can then be readily solved in the Fourier space,

$$v_k(\vec{g}) = -\mathrm{i}g^2\Omega_{ik}^{-1}(\vec{n})g_j\sigma_{ij}^0(\vec{g}) \tag{6}$$

where $\vec{g}$ is a reciprocal lattice vector, g is its length, g_j is the jth component of $\vec{g}$,

$$v_k(\vec{g}) = \int_V u_k^A(\vec{r})e^{-\mathrm{i}\vec{g}\cdot\vec{r}}d^3r \tag{7a}$$

$$\sigma_{ij}^0(\vec{g}) = \int_V \sigma_{ij}^0(\vec{r})e^{-\mathrm{i}\vec{g}\cdot\vec{r}}d^3r \tag{7b}$$

and

$$G_{ik}^{-1}(\vec{g}) = c_{ijkl}g_jg_l = g^2c_{ijkl}n_jn_l = g^2\Omega_{ik}^{-1}(\vec{n}) \tag{8}$$

with $n_i = g_i / g$.

According to equations and (7a), the real space displacement can be obtained through inverse Fourier transforms, i.e.,

$$u_k^A(\vec{r}) = \frac{1}{(2\pi)^3}\int_V v_k(\vec{g})e^{\mathrm{i}\vec{g}\cdot\vec{r}}d^3g \tag{9}$$

The next step is to find an elastic solution labeled by superscript B, i.e. $u_i^B(\vec{r})$, in an infinite plate of thickness h, satisfying the equation of equilibrium without body-force, i.e.,

$$c_{ijkl}u_{k,lj}^B = 0 \tag{10}$$

and boundary condition

$$c_{i3kl}u_{k,l}^B\Big|_{z=0,h} = c_{i3kl}(\varepsilon_{kl}^0 - u_{k,l}^A)\Big|_{z=0,h} \tag{11}$$

Equation (10) and its boundary condition (11) can be solved using a Stroh formalism of Stroh formalism of anisotropic elasticity[9,10].

Note that the macroscopic shape deformation of the film is artificially excluded due to the periodic conditions of $u_i^A(\vec{r})$ and $u_i^B(\vec{r})$. Actually, the sum of $u_i^A(\vec{r})$ and $u_i^B(\vec{r})$ gives the heterogeneous displacement $u_i^s(\vec{r})$, and the heterogeneous strain components

$$\eta_{ij}(\vec{r})=\frac{1}{2}\left(\frac{\partial u_i^s}{\partial r_j}+\frac{\partial u_j^s}{\partial r_i}\right) \tag{12}$$

As the heterogeneous strain has no macroscopic effects, we have $\int_V \eta_{ij}(\vec{r})dV=0$.

The in-plane macroscopic shape deformation can be departed into membrane strain and pure bending strain, and they are totally controlled by the external strain constraint, i.e., $\varepsilon_{\alpha\beta}^{\mathrm{M}}=\varepsilon_{\alpha\beta}^{\mathrm{memb}}+\varepsilon_{\alpha\beta}^{\mathrm{bend}}=\varepsilon_{\alpha\beta}^{a}$. Due to the two free surfaces, the membrane strain $\varepsilon_{\alpha\beta}^{\mathrm{memb}}$ would also cause a macroscopic shape deformation of the nanofilm along z direction, which can be determined according to the traction free condition, i.e., $c_{i3ij}\bar{\varepsilon}_{ij}=0$, with $\bar{\varepsilon}_{\alpha\beta}=\varepsilon_{\alpha\beta}^{\mathrm{memb}}(\alpha,\beta=1,2)$. How, it should be noted that $\bar{\varepsilon}_{i3}$ thus obtained is only part of the total shape deformation of the nanofilm. The domain structure also contributes to the macroscopic shape deformation of the nanofilm, given that the averages of the eigenstrain over the simulation cell $<\varepsilon_{i3}^0>\neq 0$. Taking into account this contribution, we have the total macroscopic shape deformation of the nanofilm as $\varepsilon_{\alpha\beta}^{\mathrm{M}}=\varepsilon_{\alpha\beta}^{a}$, $\varepsilon_{13}^{\mathrm{M}}=<\varepsilon_{13}^{0}>$, $\varepsilon_{23}^{\mathrm{M}}=<\varepsilon_{23}^{0}>$, and $\varepsilon_{33}^{M}=<\varepsilon_{33}^{0}>-c_{12}\left[(\varepsilon_{11}^{a}-<\varepsilon_{11}^{0}>)+(\varepsilon_{22}^{a}-<\varepsilon_{22}^{0}>)\right]/c_{11}$. After obtaining the macroscopic shape deformation strain, the total strain is given by $\varepsilon_{ij}=\varepsilon_{ij}^{M}+\eta_{ij}$.

3. Electric field of a nanofilm

Suppose there is no space charge inside the film. The electrostatic equilibrium

equations of the nanofilm can be described by

$$\varepsilon_{\mathrm{b}}\varphi_{,ii} = P_{i,i} \tag{13}$$

where φ being the electric potential.

For the electric field of a nanofilm under short-circuit condition, it can be also approximated by a solution satisfying periodic condition. Therefore, we can employ the same methodology to solve the electrostatic equilibrium equation as that used in solving the elastic equilibrium equations. Application of the Fourier transformation

$$\phi(\vec{g})=\int_V \varphi(\vec{r})e^{-\mathrm{i}\vec{g}\cdot\vec{r}}d^3r \tag{14a}$$

$$P_i(\vec{g})=\int_V P_i(\vec{r})e^{-\mathrm{i}\vec{g}\cdot\vec{r}}d^3r \tag{14b}$$

yields

$$g_i g_i \varepsilon_b \phi(\vec{g})=-\mathrm{i}g_i P_i(\vec{g}) \tag{15}$$

The real space potential can be obtained through inverse Fourier transforms, i.e.,

$$\phi(\vec{g})=\frac{1}{(2\pi)^3}\int_V \varphi(\vec{r})e^{-\mathrm{i}\vec{g}\cdot\vec{r}}d^3g \tag{16}$$

4. Method of writing cylindrical domain patterns into simulation cell

To write a cylindrical domain into a simulation cell, we first “pole” the simulation cell into a single domain state. This is achieved by imposing an external electric field (e.g., 10^8V/m) to the simulation cell. After a stable single domain is formed under the external electric field, we remove the external electric field and let the single domain state relax to ground state at zero-field. Due to the short circuit condition, it is found that a single domain state can be always formed through this “poling” process. The cylindrical domain patterns are then written into the simulating

cell by artificially inverting the polarization (i.e., changing the sign of P_3 component) at the written region.

5. Supplementary Tables and figures

Parameter	Value	Unit
a_1	$3.85(T-752)\times10^5$	$C^{-2}m^2N$
a_{11}	-7.3×10^7	$C^{-4}m^6N$
a_{12}	7.5×10^8	$C^{-4}m^6N$
a_{111}	2.6×10^8	$C^{-6}m^{10}N$
a_{112}	6.1×10^8	$C^{-6}m^{10}N$
a_{123}	-3.7×10^9	$C^{-6}m^{10}N$
c_{11}	1.746×10^{11}	Nm^{-2}
c_{12}	0.7937×10^{11}	Nm^{-2}
c_{44}	1.1111×10^{11}	Nm^{-2}
Q_{11}	0.089	$C^{-2}m^4$
Q_{12}	-0.026	$C^{-2}m^4$
Q_{44}	0.0675	$C^{-2}m^4$
G_{110}	1.73×10^{-10}	m^4NC^{-2}
G_{11}	G_{110}	m^4NC^{-2}
G_{12}	0	m^4NC^{-2}
G_{44}	$0.5G_{110}$	m^4NC^{-2}
G'_{44}	$0.5G_{110}$	m^4NC^{-2}
δ_i^{eff}	5×10^{-9}	m
ε_b	4.425×10^{-10}	Fm^{-1}
f_{12}	10	V

Table S1. Values of parameter used in the phase-field simulations (SI units and T in K). Values of the expansion coefficients of the Landau potential, elastic stiffness and electrostrictive coefficients are from ref. 2, the isotropic gradient coefficients are from ref. 10, the extrapolation length is from ref. 11, and the background dielectric constant is chosen according to ref. 4 and 5. The magnitude of f_{12} is a roughly estimated one due to the uncertainty of this parameter of the material[7].

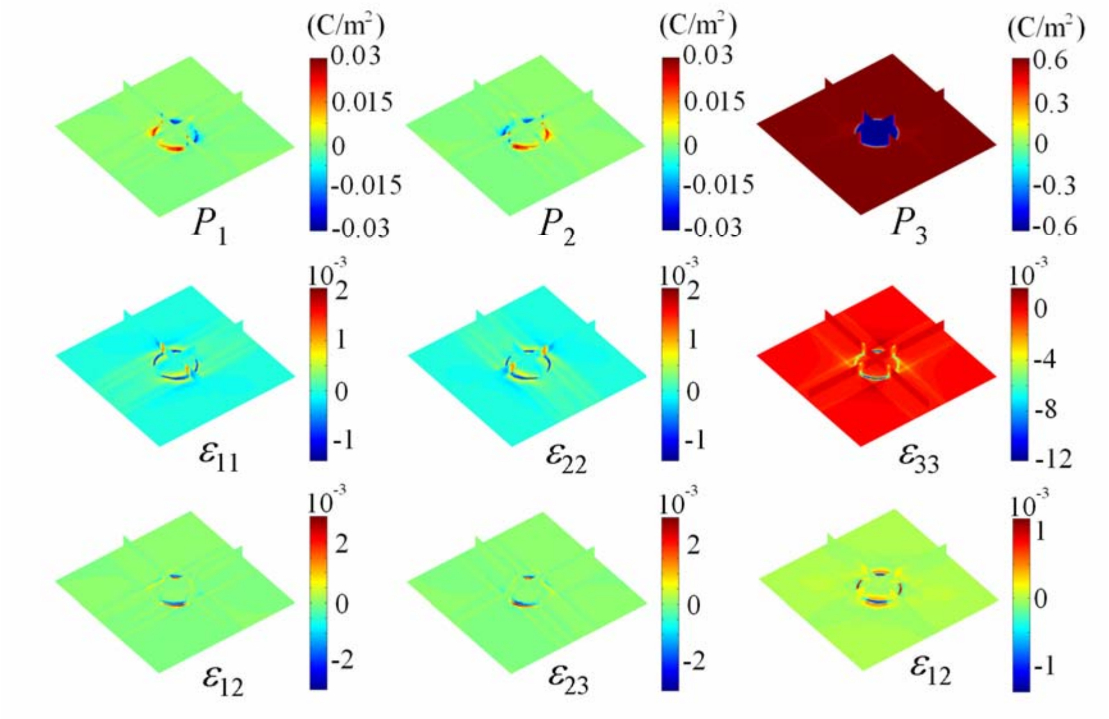

Figure S1. Three dimensional distributions of polarization field and strain field of the equilibrium domain pattern in a 128nm×128nm×8nm simulation cell written with a 16nm cylindrical domain at room temperature.

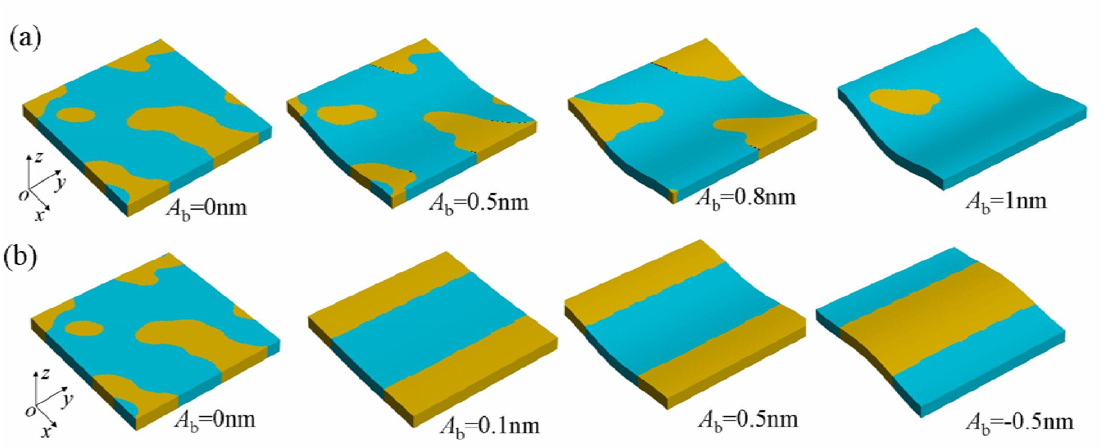

Figure S2. Strain gradient effect on the formation of domain structure in a 128nm×128nm×8nm simulation cell with an initial random polarization distribution at room temperature. Simulation results with flexoelectric field (a) switched off (b) and switched on. The simulation cell is under wavy bending with strain in form of $\varepsilon_{11}^{a} = 4\pi^2 A_b \lambda^{-2}(z-h/2)\cos(2\pi x/\lambda)$, with different wavy bending amplitude A_b and wave length $\lambda = 128\text{nm}$.

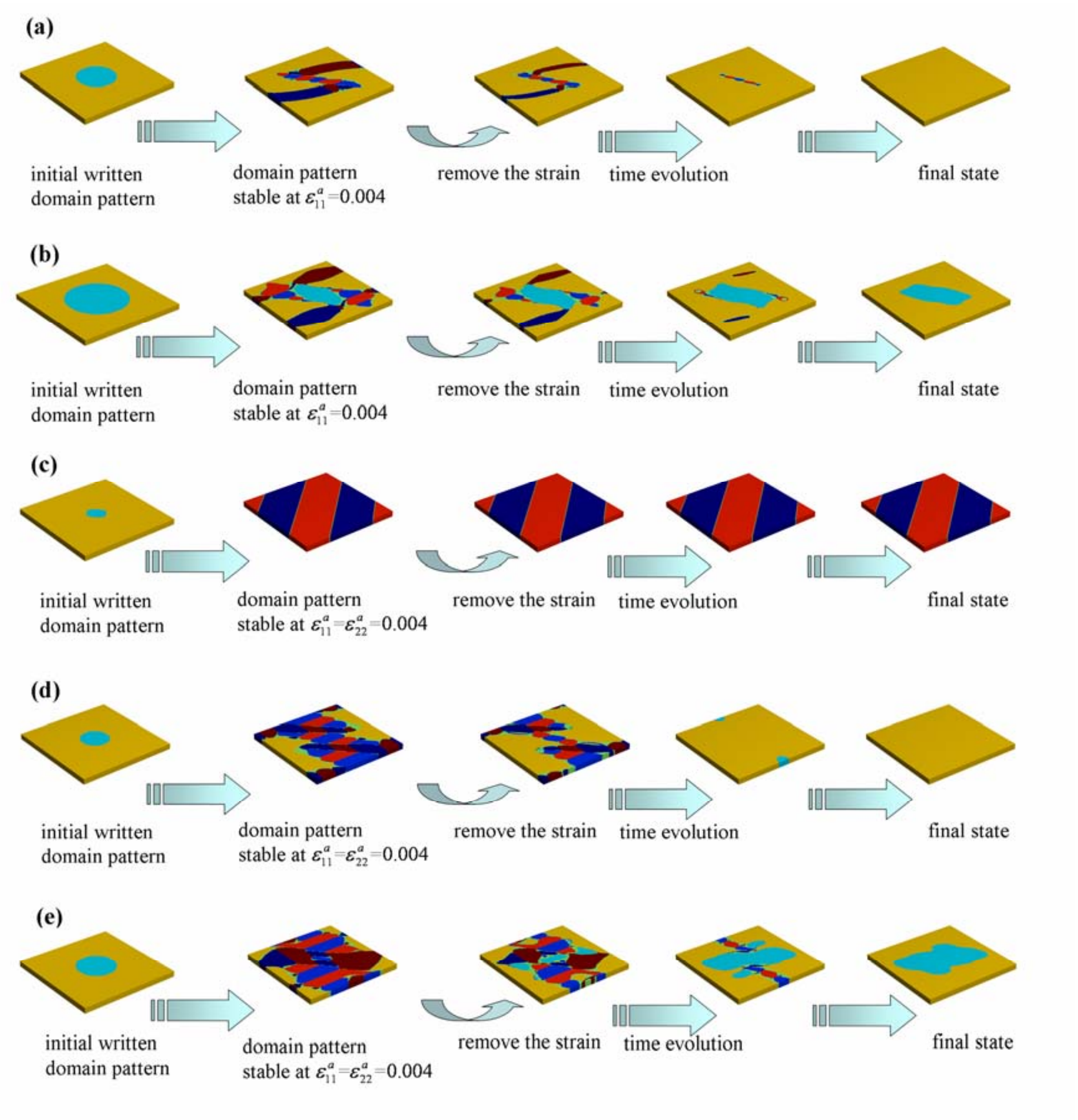

Figure S3. Typical evolution paths of domain patterns with induced *a*/*b*-domains after the strain load is off. Initial domain size (a) *r*=16nm, (b) *r*=36nm, (c) *r*=12nm, (d) *r*=16nm, and (e) *r*=24nm.

Reference

1. Haun, M. J., Furman, E., Jang, S. J., McKinstry, H. A. & Cross, L. E. Thermodynamic theory of $PbTiO_3$. *J. Appl. Phys.* **62**, 3331 (1987).

2. Pertsev, N. A., Zembilgotov, A. G. & Tagantsev, A. K. Effect of Mechanical Boundary Conditions on Phase Diagrams of Epitaxial Ferroelectric Thin Films. *Phys. Rev. Lett.* **80**, 1988 (1998).

3. Kretschmer, R. & Binder, K. Surface effects on phase transitions in ferroelectrics and dipolar magnets. *Phys. Rev. B* **20**, 1065 (1979).

4. Zheng, Y. & Woo, C. H. Thermodynamic modeling of critical properties of ferroelectric superlattices in nano-scale. *Appl. Phys. A: Mater. Sci. Process.* **97**, 617-626 (2009).

5. Woo, C. H. & Zheng, Y. Depolarization in modeling nano-scale ferroelectrics using the Landau free energy functional. *Appl. Phys. A: Mater. Sci. Process.* **91**, 59-63 (2008).

6. Landau, L. D., Lifshitz, E. M. & Pitaevskii, L. P. *Electrodynamics of continuous media*, Oxford University Press: New York, (1984).

7. Zubko, P., Catalan, G. & Tagantsev, A. K. Flexoelectric Effect in Solids. *Annu. Rev. Mater. Res*. **43**, 387 (2013).

8. Khachaturyan, A.G. Theory of structural transformations in solids. Wiley: New York, 1983.

9. Ting T. C. T. Anisotropic elasticity: theory and applications. Oxford University Press: New York, 1996.

10. Li, Y. L., Hu, S. Y., Liu, Z. K. & Chen, L. Q. Effect of substrate constraint on the stability and evolution of ferroelectric domain structures in thin films. *Acta Mater*. **50**, 395 (2002).

11. Ishikawa, K. & Uemori, T. Surface relaxation in ferroelectric perovskites. *Phys. Rev. B* **60**, 11841 (1999).
